# Analysis of the lifetime and culling reasons for AI boars

**DOI:** 10.1186/s40104-017-0179-z

**Published:** 2017-06-01

**Authors:** Damian Knecht, Anna Jankowska-Mąkosa, Kamil Duziński

**Affiliations:** Institute of Animal Breeding, Wroclaw University of Environmental and Life Sciences, Chelmonskiego 38C, 51– 630 Wroclaw, Poland

**Keywords:** AI centers, Boars, Culling, Exploitation, Management

## Abstract

**Background:**

The aim of the study was to analyze the lifetime and culling reasons for boars used in insemination centers (AI centers).

**Methods:**

The data collected from 355 culled boars from 1998 to 2013 included: age at start of semen collection, boar herd life, culling reason, daily gain and lean meat content, and number of ejaculates not meeting sales requirements after dilution. Culling reasons were divided into 7 groups: low semen value (LSV), low or lack of libido (LL), leg problems (LP), infectious diseases (ID), old age (OA), reduced demand for semen from the given boar (RD), and others (OT).

**Results:**

The most common culling reasons for boars were LSV (23.7%) and RD (22.5%). It was observed that the lowest daily gains were noted in boars culled due to OA. Boars culled due to OA and RD were maintained in production for the longest time (over 1000 d), for LSV and ID retention was about 700 d, and due to LL below 400 d. The survival probability was over 0.9 until 1.5 yr, and just over 0.2 until 4 yr. The highest relative frequency was observed in the 36^th^ and 42^nd^ mo of life (over 16%). Hazard risk analysis revealed a more than 10 times higher risk of culling in the case of LL, ID or OT, in comparison to OA.

**Conclusions:**

The results can be used as a direct point of reference for the identification of emerging problems in AI boar exploitation and the development of an appropriate culling policy in AI centers.

## Background

The reasons for culling of boars demonstrate a close relationship with the efficiency and economic profitability of operations in commercial herds and AI centers. With increasing interesting in AI, the most important role started to play AI centers. It was estimated that already 70% of Polish sows are inseminated and 99% using fresh semen, and almost 85% of this demand come from AI centers. An understanding of the reasons for culling is necessary information for the planning and rational management of such units. It should be noted that there are two main forms of boar culling, i.e. unplanned and planned. Unplanned culling (forced), i.e. culling as a result of diseases, sudden falls, behavioral problems, and lameness, clearly adversely affects productivity. In terms of productivity, Safranski [1] noted that the collection and production processes of insemination doses is dependent on a number of factors. These factors hinder the long-term accurate estimation of exploitation predispositions of boar, and thus this creates uncertainty in production. On the other hand, planned culling (decision-making) (old age, poor production results), despite the initial costs incurred, over the long term appears to be the most appropriate and cost-effective decision. Unfortunately, decisions about culling boars from a herd are still taken reluctantly by owners and are also often postponed. Only an appropriate culling schedule and herd replacement program can satisfy the essential prerequisite for the maintenance of production stability and repeatability [2].

Boars are very sensitive to abrupt changes which impair correct semen formation [3]. Preparing detailed records about culling within a specified time (e.g. annual balance) allows for the identification of risks to the boar population, especially in terms of possible diseases, disorders, and behavioral problems [4]. It is important for AI centers to have an active population of boars that produce appropriate ejaculates with high quality and quantity parameters [5], which is also connected with proper preparation of doses [6].

Analysis of culling reasons enables a clear increase in the production efficiency of AI units. Although it might seem that in terms of the profitability of pig production all avenues have already been investigated, research into the control, improvement and acceleration of time required to obtain effective production capacity of boars is still valid. Progress in the production results of modern piggeries may be supported by AI [7]. The development of AI has been made possible due to the constant production of high value semen doses [8]. Smital [9] stated that the economic effectiveness of AI centers is closely dependent on the productivity of boars during exploitation, and is limited in the highest degree by the construction of the testicles, libido, and physical activity (limb, spine defects). However, according to the results of previous studies, the problems of sperm quality are not the only reason for boar culling [3, 4, 10, 11], and this has necessitated a deeper analysis of all possible causes.

Given the above consideration, it can be clearly stated that the choice of boar for AI centers and their accurate monitoring during exploitation are very important issues. Once selected, boars should remain within the active productive population for as long as possible, compensating costs and generating incomes for AI centers [4]. Recouperation of the maintenance and exploitation costs of AI boar stations usually occurs after 2–3 yrs of a boar’s life [12]. Only the direct identification of culling reasons can skillfully define the problems faced in herds of boars maintained at AI centers. Unfortunately, this subject is still overlooked in global scientific publications. Most articles focus on an analysis of culling reasons and lifetime of sows and only some concern boars, but these mostly relate to those on breeding farms [3, 4, 10, 11] and not AI boars. Therefore, the aim of our study was to analyze lifetime and culling reasons for boars used in AI center.

## Methods

### Experimental location and animals

The study was carried out between 1998–2013 at the Boar Exploitation Station in Częstochowa. The study population included 355 culled boars, whose histories of exploitation were followed from birth until death. The presented population was representative in proportion to the most common breed components used for AI in Poland, such as: Polish Landrace, Polish Large White, Duroc, Pietrain, Hampshire, Duroc × Pietrain, Hampshire × Pietrain, Duroc × Hampshire. The collected data from 1998 to 2013 included: age at start of semen collection, boar herd life, culling reason, daily gain and lean meat content (evaluated before purchase of boar), and number of ejaculates not meeting the requirements of sales after dilution (ejaculates incompatible with requirements). Reasons for culling were divided into 7 groups: low semen value (LSV), low or lack of libido (LL), leg problems (LP), infectious diseases (ID), old age (OA), reduced demand for semen from the given boar (RD), and others (OT). The whole population of boars was divided into experimental groups based on the reason for culling.

Decision process for unplanned and planned culling were different. The structure of unplanned culling were simple and based on the first observation of boars by employees receiving ejaculates. Information were directed to the supervisors and the director, the director usually took the sole decision about unplanned culling. In the case of the planned culling decision-making process was more complex. The long-term replacement plan was developed and approved by the owner and the supervisory board. After all, plan was also constantly being upgraded under the flowing marketing information and the demand for a certain product. On this basis planned culling were made.

Therefore, there were finally 7 groups of culling reasons (LSV, LL, LP, ID, OA, MU, OT). In subsequent years the ratios of culling reasons were similar therefore, the sample period was treated jointly. The overall characteristics of the study population are presented in Table 1.

**Table 1:** The overall characteristics of the study population of boars (*n* = 355)

Trait	Mean	SD	Min	Max
Age of semen collection entry, d	259.74	33.51	201	458
Boar herd life, d	835.57	503.5	21	2350
Daily gain, g	775.81	94.32	554	1170
Lean meat content, %	61.34	1.94	55	67
Ejaculates incompatible with requirements, n	3.36	5.49	0	37

*SD* standard deviation, *Min* minimum, *Max* maximum

### Daily gain and lean meat content

The assessment of daily gain and lean meat content, before purchase of the boar, was made between d 170 and 210 of life. Daily gains were calculated by dividing the body weight of young boars (during assessment time) by the age on the assessment day. The measurements of the animals for the estimation of daily gains were made using a Mensor WM150P1 electronic scales. Daily gains were standardized to 180 d using a model developed for standardization in accordance with the methodology of Mucha and Różycki [13], in order to reduce age differences during assessment. The percentage of meat content in carcasses was estimated intravitally on the basis of two backfat thickness (points P2 and P4) and lion eye height (point P4) measurements. The measurements were made using a PIGLOG 105 (SFK) ultrasonic device, positioned behind the last rib (between the thoracic and lumbar vertebrae), 3 cm off the midline (point P2) and 8 cm off the midline (point P4). To increase the accuracy of the assessment, standardization of traits was performed to 110 kg body weight. The measurement values were inserted into a specially developed equation [14], which allowed an estimation of the intravital proportion of meat in the carcass on the assessment day. The results for meat content were also standardized to d 180 [13].

### Boars performance

Before the start of semen collection, all boars were held in quarantine, the length of which was approximately 37.24 ± 5.02 d. During quarantine semen was collected once a wk just to observation only and not for insemination. Additionally, all boars were exploited in the same manner, developed and adopted according to the methodology of the AI station. Ejaculates were collected by masturbation via the manual method using a container with a filter. The gelatinous fraction was separated. Immediately after collection, the volume of semen was measured using a scalar cylinder. The concentration of spermatozoa was evaluated using a SpermaCue device, Model 12300/0500 (Minitube International, Verona, USA). Based on the semen volume and spermatozoa concentration, the total number of spermatozoa in the ejaculate was calculated. Semen dilution was effected using the same semen extender. Boars until 10 mon gave ejaculates once a week, at the age of 10–14 mon this was three times in two week and from 15 mon twice a week. The average annual replacement rate was 49.8%. Ejaculates were classified as normal for further dilution, when the following requirements were met: color from gray to milky white, flavor specific, lack of foreign admixtures, more than 70% of progressive motile sperm cells, pH 7.0–7.9, morphological abnormality changes to 15% (5% primary, 10% secondary). Insemination doses of 80 mL contained a constant 2.8 × 10^9^ spermatozoa. Semen was stored at 15 °C for not longer than 48 h.

### Housing and feeding

Boars were single-housed and maintained in accordance with the principles of animal welfare [15]. Each individual pen area was 8 m^2^/boar. Boars were kept on a solid concrete floor, which was covered with straw. The air temperature in all the boar pens was close to 15 °C (min 12 °C, max 20 °C). Relative humidity was close to 75% (min 65%, max 85%). The air circulation inside the building was equal to 0.15 m/s in Winter and 0.20 m/s in Summer. Preventive care and vaccination was carried out regularly in accordance with the methodology of the unit. The microclimate of the area and ventilation were controlled by computer. Over the whole study period, boars were fed the same all-mash mixture, dosed according to the recommended nutrition standard for boars, with permanent access to water (Table 2).

**Table 2:** The nutritional value of 1 kg of compound feed for boars

Item	Value
Dry matter, g	887
Metabolizable energy, MJ	12.5
Crude protein, g	169.63
Fat, g	41.98
Crude fiber, g	59.58
Lysine, g	9.38
Methionine + cystine, g	6.14
Threonine, g	5.9
Tryptophan, g	1.8
Valine, g	5.9
Isoleucine, g	4.5
Ca, g	7.13
P total, g	5.6
Na, g	1.8
Vitamin A, IU	15,000
Vitamin D_3_, IU	2000
Vitamin E, mg	100
Vitamin C, mg	1000
Vitamin K, mg	5
Vitamin B_1_, mg	2
Vitamin B_2_, mg	7
Vitamin B_6_, mg	4
Vitamin B_12_, μg	30
Biotin, μg	400
Folic acid, mg	4
Niacin, mg	30
Pantothenic acid, mg	15
Choline, mg	1185
Mn, mg	50
Fe, mg	145
I, mg	1
Zn, mg	100
Cu, mg	20
Co, μg	500
Se, μg	300

### Statistical analysis

The data was analyzed using the STATISTICA (2014) statistical program. The values in the tables are arithmetical means ($\overline{x}$) and standard deviations (SD). A Chi-squared test was used to examine the significance of differences for the relative frequency (%) of boars removed from the removal groups. Other permanent collected data were checked for normality with the Kolmogorov-Smirnov (K-S) test with the Lilliefors correction. In addition, the Brown-Forsythe test (B-F) determined whether the distributions of the variables had the same variance. An advanced mixed model using the GLM procedure was used and an analysis of variance was conducted for factorial designs to determine the effect of tested parameters by the culling reason. The significance of differences was calculated on the basis of Tukey’s multiple range test. The levels of significance of differences were given conventionally: significant 0.01 < *P* ≤ 0.05 and highly significant *P* ≤ 0.01. Pearson’s correlation coefficient (r) was calculated between daily gains, lean meat content and age at start of semen collection entry, and boar herd life. Survival analysis was performed using the Cox proportional hazard model. The time variable was defined as age in months from birth. The year effect in herd was included in the analysis. The Cox proportional hazard model expressed risk during t for the tested independent variable system and was expressed by the equation:

$$ \mathsf{h}(t) = {h}_{\mathsf{0}}{\mathsf{e}}^{\mathsf{\beta iXi}} $$

where: h(t) was the risk at a given time interval, h_0_ the base risk (the risk obtained if there were no risk factors in the model), and β_i_ the regression coefficient for the i-th removal reasons. The hazard ratio (HR) for the situation when the risk factor X is present versus the situation when the risk factor X is absent was calculated as:

$$ \mathrm{H}\mathrm{R} = \frac{h\left( t, x=1\right)}{h\left( t, x=0\right)}={\mathsf{e}}^{\mathsf{\beta iXi}} $$

## Results

The results of the assessment of the selected parameters of boar performance, depending on the culling reasons, are presented in Table 3. The most common culling reasons were LSV, followed closely by RD. Differences amounting to only 1.2% (*P* > 0.05) were noted between these reasons. However, of these the two most common culling reasons demonstrated statistically significant differences with other reasons, i.e. LP (*P* ≤ 0.05) and ID, OA, LL, OT (*P* ≤ 0.01). Boars culled due to LL were characterized by the highest age at the start of semen collection. The difference from the youngest age at the start of semen collection observed for boars culled due to ID was over half mo (*P* ≤ 0.05). The longest boar herd life expectancies were noted for boars culled due to OA, and differences with other groups were statistically proven at the level of *P* ≤ 0.01. Boar herd life expectancy in excess of 1000 d were achieved by boars culled due to RD. Boars with LSV and ID were held in production for over 700 d, and the shortest period was noted for boars with LL, i.e. less than 400 d. It was observed that the lowest daily gains were noted in boars culled due to OA, and differences with other reasons were at a level from 53.32 g to 68.14 g (*P* ≤ 0.05), with the exception of RD (*P* > 0.05). Similar observations have been made for the lean meat content parameter and differences for OA were statistically confirmed with LSV, ID and OT (*P* ≤ 0.05). The highest number of ejaculates incompatible with sales requirements after dilution were recorded in boars culled due to ID and the lowest were for LL, RD and OT boars (all below 3; *P* ≤ 0.01).

**Table 3:** Selected parameters of boar performance by culling reasons (mean ± SD)

Item	LSV	LL	LP	ID	OA	RD	OT
Proportionate rate, %	23.7^Aa^	9.3^B^	14.9^b^	9.6^B^	9.3^B^	22.5^Aa^	10.7^B^
Age of semen collection entry, d	259 ±37	267 ±31^b^	261 ±32	251 ±29^a^	258 ±48	261 ±27	259 ±31
Boar herd life, d	738 ±463^B^	399 ±321^A^	695 ±381^AB^	759 ±415^B^	1605 ±433^D^	1105 ±309^C^	460 ±276^AB^
Daily gain, g	786.13 ±86.89^a^	788.55 ±129.72^a^	783.91 ±107.62^a^	797.29 ±83.4^a^	729.15 ±94.03^b^	761.3 ±73.41	782.47 ±93.02^a^
Lean meat content, %	61.62 ±1.82^a^	61.29 ±2.32	61.27 ±1.44	61.65 ±1.77^a^	60.43 ±2.2^b^	61.25 ±1.95	61.54 ±2.18^a^
Ejaculates incompatible with requirements, n	3.42 ±5.72	1.85 ±2.76^A^	3.92 ±5.58	5.79 ±8.64^B^	3.73 ±5.68	2.54 ±4.66^A^	2.97 ±3.74^A^

*LSV* low semen value, *LL* low or lack of libido, *LP* leg problems, *ID* infectious diseases, *OA* old age, *RD* reduced demand for semen of the given boar, *OT* others

^a,b^ – in the same row signifies statistically significant differences between reasons of culling, with *P* ≤ 0.05

^A,B,C,D^ – in the same row signifies statistically significant differences between reasons of culling, with *P* ≤ 0.01

**Fig. 1:**
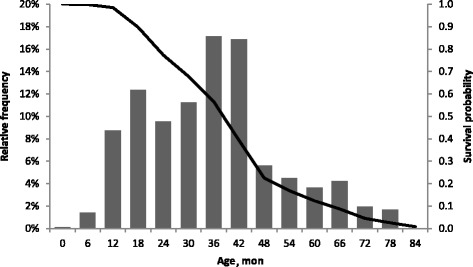
Survival probability of boars in the AI station and relative frequency (column) of boar removal by age in mon

The estimated survival probability of boars in the AI station and the relative frequency of boar removal in each month are presented in Fig. 1. A survival probability for boars of over 0.9 was noted until 1.5 yr. A drastic drop in the probability was observed to the age of 4 yr, achieving a value a little over 0.2. From this point, survival probability gently fell (expired) to 7 yr, when there was a complete replacement of the herd. The highest relative frequencies were observed at the 36^th^ and 42^nd^ month (over 16%). Indicators of relative frequency over 10% were also noted in the 18^th^ and 30^th^ mo of life.

**Fig. 2:**
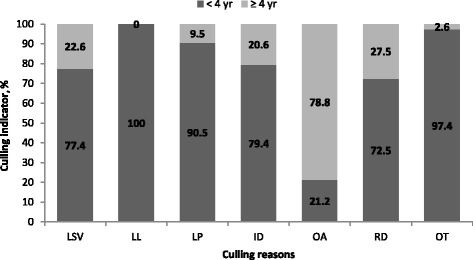
The percentage of boars culled before and after 4 yr, by culling reasons. LSV- low semen value, LL- low or lack of libido, LP- leg problems, ID- infection diseases, OA- old age, RD- reduced demand for semen of the given boar, OT- others

Figure 2 shows the percentage of boars culled both below and above 4 yr, depending on the reason. It was noted that all boars with LL were culled by 4 yr. Equally large culling rates by 4 yr were reported for boars with OT and LP. More than 70% of boars from the ID, LSV or RD groups were culled by 4 yr. The highest levels of culling over 4 yr were obtained for the OA group.

The hazard analysis for culled boars depending on the reason is presented in Table 4. The highest hazard ratios for culling were achieved for OT, LL and ID. The lowest hazard ratios (i.e. 3.08 to 4.38) were noted for LSV, RD and LP. The highest parameter values were observed for LL and OT, and the lowest negative values occurred for RD.

**Table 4:** Hazard analysis for culled boars depending on the reason

Item	Parameters	Standard error	Hazard ratio	Confidence interval
LSV	0.031	0.112	3.08	1.86; 5.01
LL	1.063	0.143	10.47	5.45; 20.1
LP	0.191	0.131	4.38	2.49; 7.72
ID	1.002	0.157	10.42	5.79; 18.75
OA	0	0	1	-----
RD	−0.575	0.113	3.29	2.01; 5.38
OT	1.055	0.156	16.81	9.51; 29.73

*LSV* low semen value, *LL* low or lack of libido, *LP* leg problems, *ID* infectious diseases, *OA* old age, *RD* reduced demand for semen of the given boar, *OT* others

Table 5 shows correlation coefficients of selected parameters related to culling reasons. Statistically confirmed (*P* ≤ 0.05) positive correlations between daily gain and age at the start of semen collection were observed for boars from OA, ID and LSV groups. The remaining statistically significant positive correlations were reported for OA between daily gain and boar herd life expectancy, and also lean meat content and age of semen collection entry. Additionally, for LP boars positive correlations were calculated between lean meat content and age at the start of semen collection or boar herd life expectancy (*P* ≤ 0.05). Three negative correlations were found (*P* < 0.05). Two of these were noted for boars culled due to LL between daily gains and boar herd life expectancy, and also lean meat content and boar herd life expectancy. The last negative correlations were observed between lean meat content and age at start of semen collection for RD boars.

**Table 5:** Correlation coefficients for selected parameters by culling reasons

Item	LSV	LL	LP	ID	OA	RD	OT
Daily gain/age of semen collection entry	0.26*	−0.07	0.09	0.21*	0.32*	−0.16	0.14
Daily gain/boar herd life	0.12	−0.44*	0.03	0.05	0.28*	0.08	−0.01
Lean meat content/age of semen collection entry	0.08	0.17	0.34*	−0.14	0.23*	−0.34*	−0.11
Lean meat content/boar herd life	0.16	−0.49*	0.31*	0.18	0.05	0.01	0.13

*LSV* low semen value, *LL* low or lack of libido, *LP* leg problems, *ID* infectious diseases, *OA* old age, *RD* reduced demand for semen of the given boar, *OT* others

* – correlation statistically significant, with *P* ≤ 0.05

## Discussion

The results presented here provide valuable information on the culling reasons for boars. A proportionate rate of culling reasons is a useful indicator of the strength of reasons and helps prioritize the improvement of a herd [10]. Observed culling of boars due to LSV and LL combined amounted to 33%, which was not as high as in the results of previous studies, which amounted to 19–25% [4, 11]. Such discrepancies could be explained by the higher culling ratio in experimental units and farms using AI, for which the requirements in this regard are much greater [10]. Additionally, current capabilities for the accurate microscopic assessment of ejaculates contribute to changes in the classification of culling reasons especially semen value [11]. The more precise the characterization of culling reasons, the more accurate the analysis of the herd and the problems appearing therein and also the expected herd life of boars.

The average age at the start of semen collection and boar herd life approached the results obtained in piggery farms [4, 11], and was also much longer than in breeding herds from the early 1990s [10].

In contrast to the study of Koketsu and Sasaki [11], age at the start of semen collection had an effect on the reason for culling. Boars culled due to LL were the latest to begin semen collection. This is probably because the jump reflex was poorly noticeable, and this consequently led to a delay in the introduction of boars to ménage and a later analysis of the reason for culling. Libido is determined by many factors, including the level of circulating testosterone in the male body [16]. AI stations do not typically prefer to maintain boars demonstrating low libido, because it impacts upon operational costs and therefore such boars are eliminated from an active herd as soon as possible. Berger and Conley [17] even stated that boars with low libido and demonstrating a low ability of sperm to fertilize are useless for the AI industry, as are diseased animals.

Boars with diagnosed infectious diseases were characterized by the youngest age at start of semen collection. A longer period of quarantine, acclimatization and isolation before starting semen collection is recommended to reduce the occurrence of new pathogens and deaths of animals as a result of infection [18]. In our study, quarantine time was unified; therefore, in the case of the analyzed place, and the age at the start of semen collection could be extended by a few days without any negative effect on the remaining culling reasons. Such preventive action can lead to a reduction in boars culled due to ID.

Herd management in AI stations is significant, especially health status. Health reasons for boar culling include not only infectious diseases but also limb problems or unexpected falls. The health status of boars is a complex factor and results not only from veterinary care but also nutrition, management, exploitation, maintenance conditions or individual characteristics [19]. During culling analysis it is reasonable to separate the major determinants of health, i.e. infectious diseases and limb problems. The high levels of culling due to LP in our study remained close to results from a study conducted by D’Allaire and Leman [10], although other authors have noted both higher [3] and lower values [4, 11]. Regardless of the research, herd life expectancy of boars was similar. Important for the diagnosis of locomotor problems in boars is rapid preventive and curative action, because these disorders are classified as being painful for animals [15].

Due to the use of pigs for high quality pork, particular importance during production parameter analysis should be placed on daily gains and lean meat content [20]. It was observed in our study that boars with higher daily gains and lean meat content had a greater predisposition to infectious diseases. On the other hand, boars culled due to OA were characterized by the lowest daily gains and lean meat content. It can be concluded, therefore, that improvement of AI boars in terms of daily gains and lean meat content is justified only to a certain point. Our earlier (unpublished) results have shown that differences between semen parameters for boars with daily gains 700–750 g and 750–800 g are almost the same, are differences between lean meat content. Such precise analyses help us to conclude, that selection of AI boars with lower daily gains allows for longer boar herd life expectancy and culling herds with greater size due to old age. Wolf [21] stated that production traits (daily gains and lean meat content) have a negligible impact on semen characteristics, but play an important role in boar exploitation, which is especially important for AI centers. Confirmation of this thesis may be presented in our study in the form of the statistically confirmed correlations for exploitation parameters between daily gain and lean meat content. Inheritance of important production traits is essential, particularly in AI.

A significant emphasis is placed on the impact of sires on offspring in all currently existing insemination programs, where an extremely large number of offspring are obtained from each sire [22]. The importance of the proper and efficient functioning of AI stations can be seen in the sale of insemination portions consistent with the expectations of customers [23]. Therefore, great emphasis should be placed on the selection of a boar to achieve profitability of purchase. The identification of the market factor (RD) as one of the main culling reasons clearly shows that artificial insemination has become so popular, and competition in the market so large that in order to maintain the stability of a company adaption to customer requirements and market flexibility is required. As presented in the results, the culling of boars for reasons including market factors (reduced demand) represents an important element in a reasonable culling policy for AI boars and was ranked second. Previous studies on culling boars did not characterize this factor alone and the factor was probably included in the “other reasons” category. Most recent work in this area seems to indicate a higher proportion of culling for “other reasons” (c. 20%) [4, 11] in comparison with our results. Selection of boars is mainly based on the selected parameters of growth and carcass traits, with minimal emphasis on production traits [5]. However, AI stations should not be limited only to selection of boars due to the best production traits for pig producers, but must also take into account factors that affect the individuals’ performance, i.e. the construction, temperament, quantity and quality of ejaculate [24]. A company operating on the market and focused on profitability has to meet the needs of customers. The constantly growing popularity of AI contributes to problems with fulfilling needs in terms of the appropriate quality of insemination portions [25]. The best solution is, therefore, to agree upon a compromise choice. If there is excessive pressure to choose traits favoring the AI stations, then market traits may be overlooked. A lack of demand for the offered insemination portions from a specific boar means its maintenance is unjustified. Culling for economic reasons may also take place in the case of new boars of the same genotype which are characterized by much better production parameters from their predecessors. In such a situation a decrease in demand for insemination portions from predecessor may be observed and this raises questions concerning its further use or culling, because the economy affects the company’s balance sheet. Production farms are located in a particular economic environment, thus economic culling reasons should be taken into account in research. Hence, it was fully justified for our study to determine the reduced demand for semen of a boar to be a reason which closely affects the survival analysis of the herd.

Survival analysis is the recommended method for the study of boar stayability in herds [26]. Our results are similar to those in Segura-Correa et al. [4] but were only observed in farm A. Other farms in the study by these authors were characterized by much lower survival probability. On the other hand, in research conducted by Koketsu and Sasaki [11] a survival function with a similar shape was shown, although with increasing age the survival probability was higher in our study. However, boar herd life expectancy in high-performing herds and in our AI centre ended after 78 mon (6.5 yr). It is believed that culling boars from a high production herd should take place no later than the age of 6 yr. Nevertheless, it is suggested that culling of boars with low semen values and andrology-fertility indicators significantly below expectations should occur as soon as possible [5]. Culling in the AI center fits the shape of the survival curve. A higher share of culling due to old age proves the probity of the decisions concerning choosing boar and proper management in the unit. The critical point in the economic viability of boars exploitation is the age of 2–3 yrs [12], which in the case of our own research has been confirmed by relative frequency. Additionally, a detailed distribution of culling reasons both before and above 4 yr has been presented to characterize the problems concerning the direct contribution of boars to AI centre profitability.

Analyzing the above-mentioned distribution, the best solution would be quick culling of diseased boars, and those with reduced libido and low sperm quality. However, sometimes individuals suffer from infectious diseases or limb problems in later years, because the disease can appear in all ages and is a time-independent parameter. In the same regard, low semen quality should be considered, because despite the development of boars producing semen with more favorable traits [24, 27], this problem can also still affect boars with a long history of exploitation and at an advanced productive age. The most unpredictable culling reason for boars to be noted from the graph is the market factor (RD), because reduced customer demand for specific semen may result from the appearance of better individuals or arising trends and fashions in pig production.

The hazard function focuses on the occurrence of an event and reflects the instantaneous potential at the time of the event. An important feature of the hazard function is the reference to a specific time [28]. In our study, the hazard risk ratio described the occurrence of boar culling in comparison with the culling of a boar due to its age. A hazard risk higher than 1 indicates an increased risk of culling boars from the active herd in comparison with culling due to age. A hazard risk ratio below 1 indicates a lower risk of culling. Therefore, one can see that there was a more than 10-fold increased risk of boar culling in the case of LL, ID or OT compared to OA. This indicates the significance of these factors in shaping the overall predictability of possible problems in a herd of boars.

## Conclusions

An understanding of culling reasons contributes to the ability to identify problems relating to AI center functioning. The results of the above study may be used as a direct point to develop appropriate strategies for boar culling in AI centers. An effective program of culling affects the economic viability of centers. A too frequent exchange of individuals and a large share of young boars negatively affects the cost of production and the health status of the herd. A large share of culling due to age, and a low rate in the case of diseases, hormonal disorders and quality of sperm provide a good selection of material production and careful management of the unit. An example of good practice is also the separate classification of economic reasons for culling (e.g. reduced demand for semen of the boar), because these reflect the changing preference of customers and the relevancy of products offered on the market. Each decision about boar culling, regardless of the reason, is difficult but necessary. However, if made at the right time and based on relevant observations, such decisions enable the preservation of the liquidity and profitability of AI centers.

## Abbreviations

AI: Artificial insemination
GLM: General linear model
HR: Hazard ratio
ID: Infectious diseases
LL: Low or lack of libido
LP: Leg problems
LSV: Low semen value
OA: Old age
OT: Others
RD: Reduced demand
SD: Standard deviation
